# Remarks on Manual Delivery of the Placenta

**Published:** 1849-08

**Authors:** R. A. Richardson


ART. VI.—*Remarks on manual delivery of the Placenta.* By R. A. Richardson, M. D.

Brooklyn, July, 1849.

Mr. Editor—*Dear Sir:* The enclosed was written in January last, and with the intention of sending it for publication, but thinking that this mode was practiced only by ourselves, and it being so much at variance with the instructions given us by our best obstetrical writers, I had, on more mature deliberation, concluded not to send it, and had not ventured to mention it to my medical brethren even; but on reading an article written by J. Byrd Smith, M. D., of Cincinnati, and the Editor of the Buffalo Medical Journal, and coinciding with the views therein expressed, I have decided to forward it, and if you think it worthy a place in your excellent Journal, you are at liberty to publish it.

Yours in haste,

R. A. RICHARDSON.

---

*To the Editor of the Buffalo Medical Journal and Monthly Review.*

Dear Sir:—I wish to make a few remarks on midwifery, as practised by myself. And, to come to the matter at *once,* when the proper time arrives, i. e., while the fœtus is being expelled from the uterus, and as the fundus becomes emptied, I make firm, but steady pressure over this part of the uterus, and by this manœuvre, if conducted properly and with care, an efficient and uniform contraction of this organ is insured from above downwards, and in 12 out of 15 cases in which I have practiced in this way, the placenta has been separated from the uterus, and in 5 out of the 12 cases, was expelled with the same pains that expelled the child. I grasp the uterus with both hands, and under a firm but moderate pressure, the uterus will soon be felt to contract and recede into the pelvis, and the delivery of the placenta usually accomplished. But, if in this way the placenta is not expelled, and being detached from the uterus, I seize the funis, follow it up to its attachment to the placenta, introducing a finger into its structure, and slowly withdrawing the hand, the placenta follows.

Very little hemorrhage has ever followed when I have proceeded in this way, and much anxiety and apprehension of danger by the patient is obviated, and the condition of the patient rendered more comfortable, and with much less delay than when spontaneous expulsion is waited for. This, by some may be termed "meddlesome midwifery," but certainly I cannot call to mind a single case in which hemorrhage has occurred to the extent to create alarm or even anxiety.

After the delivery of the child, when hemorrhage occurs to an alarming extent, we are instructed by obstetrical writers to introduce the hand and remove the placenta with as little delay as possible, at the same time causing "friction" to be made over the region of the uterus to produce contraction. Now, the end to be obtained in such cases, seems to be the permanent contraction of the uterus, and thus checking the hemorrhage.

This being the case, why may we not accomplish the same object in the manner above described, and remove the danger of hemorrhage, prevent "vexatious delays," and render the patient in a degree comfortable, and that, too, in a comparatively short length of time.

Jefferson, January 1, 1849. Yours, &c., R. A. RICHARDSON.

P. S.—This mode I term manual delivery of the placenta.

